# Reliability and validity of a Japanese version of the Brunnsviken Brief Quality of Life Scale

**DOI:** 10.1002/pcn5.170

**Published:** 2024-02-04

**Authors:** Sayo Hamatani, Kazuki Matsumoto, Philip Lindner, Eiji Shimizu, Yoshifumi Mizuno, Gerhard Andersson

**Affiliations:** ^1^ Research Center for Child Mental Development University of Fukui Fukui Japan; ^2^ Division of Developmental Higher Brain Functions, United Graduate School of Child Development University of Fukui Fukui Japan; ^3^ Department of Child and Adolescent Psychological Medicine University of Fukui Hospital Fukui Japan; ^4^ Research Center for Child Mental Development Chiba University Chiba Japan; ^5^ Division of Clinical Psychology, Kagoshima University Hospital, Research and Education Assembly Medical and Dental Sciences Area Kagoshima University Kagoshima Japan; ^6^ Department of Clinical Neuroscience, Centre for Psychiatry Research, Karolinska Institute, & Stockholm Healthcare Services Region Stockholm Stockholm Sweden; ^7^ Department of Behavioural Sciences and Learning Linköping University Linköping Sweden; ^8^ Department of Biomedical and Clinical Sciences Linköping University Linköping Sweden; ^9^ Department of Clinical Neuroscience Karolinska Institute Stockholm Sweden

**Keywords:** life satisfaction, psychometrics, quality of life, self‐rating scale, subjective well‐being

## Abstract

**Aim:**

The Brunnsviken Brief Quality of Life Scale (BBQ) is a popular quality of life measure, indexing satisfaction with and importance of six empirically selected life domains. Whether these domains hold cross‐cultural validity remains largely unexplored. The current study developed and psychometrically validated a Japanese version of the BBQ (BBQ‐J).

**Methods:**

Data were collected from 637 Japanese individuals aged between 20 and 87 years. We used *t*‐tests, Pearson product‐rate correlation coefficients, a reliability analysis, a confirmatory factor analysis, and an exploratory factor analysis to analyze the data, with 637 participants in all analyses.

**Results:**

There were no statistically significant gender differences on the BBQ‐J. Confirmatory factor analysis of the BBQ‐J revealed a 1‐factor structure with six items. Convergent validity was confirmed by its association with life satisfaction, and negative convergent validity was confirmed by its negative correlation with depressive symptoms. Cronbach's alpha of the BBQ‐J showed high internal consistency.

**Conclusion:**

Similar to the original version, the Japanese version of the BBQ is a valid and reliable self‐administered measure of subjective quality of life for use in clinical and research settings.

## Graphical abstract

The Brunnsviken Brief Quality of Life Scale (BBQ) consists of six psychosocial indicators that are directly linked to a person's well‐being: leisure time, view on life, creativity, learning, friends and friendship, and view of self. This study demonstrated that the Japanese version of the BBQ has single factor structure with six items, and is a valid and reliable self‐administered measure of subjective quality of life for use in clinical and research settings.

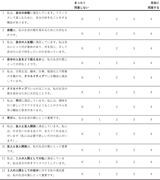

## INTRODUCTION

The World Health Organization defines quality of life (QOL) as “an individual's perception of their position in life in the context of the culture in which they live and in relation to their goals, expectations, standards, and concerns.”^1^ In recent years, QOL has been considered a crucial indicator when surveying the general population's health, along with being established as an outcome of symptoms.^2^, ^3^ In particular, subjective QOL reflects one's overall perception of and satisfaction with life situations.^4^ In 2016, Lindner et al.^5^ developed the Brunnsviken Brief Quality of Life Scale (BBQ) to measure subjective QOL, operationalized as the satisfaction with different life domains, weighted by their perceived importance. Designed to be unifactorial, the scale covers six life domains empirically shown to be of importance for a singular QOL construct. It consists of six psychosocial indicators that are directly linked to a person's well‐being: leisure time, view on life, creativity, learning, friends and friendship, and view of self. The BBQ has been found to have good internal consistency, reliability, and convergent validity and is a valid and reliable measure of subjective QOL for use in clinical and research settings. Further, it is currently available in more than 30 languages and freely downloadable at www.bbqscale.com.

Several other self‐administered scales have been developed to measure QOL. These include the medical outcomes study 36‐Item Short‐Form Survey (SF‐36),^6^ EuroQol 5 Dimension,^7^ and World Health Organization QOL.^8^ These measures assess “health‐related quality of life (HRQOL).”^9^ HRQOL is an indicator of health status, helps improve health, and is used primarily in medical settings. The two most basic components of HRQOL are subjective sense of health and daily functioning.^10^ HRQOL does not take into account psychosocial factors, such as life satisfaction or how well one gets along with one's friends, apart from aspects of functioning. Thus, the BBQ probably fares better than HRQOL in measuring how satisfied one is with one's life or how positively one views one's life. Further, HRQOL involves the “absence‐of‐disease” approach, and the abovementioned scales are not enough for use in healthy subjects when measuring subjective QOL. However, a greater degree of life satisfaction promotes habits that help people lead healthier lives, such as controlling calories, exercising, indulging in leisure activities, having good interpersonal relationships, and low use of substances.^11^ Consequently, people who perceive a greater degree of subjective well‐being might have a longer life expectancy,^12^ a lower risk of heart disease and stroke, and faster recovery from illness.^11^ Overall, subjective QOL is a vital predictor of overall health and wellness in the short as well as long run. In other words, the BBQ is also useful in measuring the subjective QOL of continuously healthy individuals.

Although, there are also some studies published using the BBQ,^13^, ^14^, ^15^ a Japanese version of the BBQ (BBQ‐J) has not yet been developed. The original BBQ was developed in Scandinavia (Sweden), a region with cultural differences from East Asia, particularly Japan. The six life domains covered by the BBQ were selected based on a factor analysis of Scandinavia (Swedish) QOL ratings. It was uncertain whether these same six life domains would collectively provide a valid QOL measure in a Japanese context, given the cultural differences. Generally, the construct of QOL appears to be quite similar in different cultures, but it has also been reported that different cultures respond differently to specific items related to QOL.^16^, ^17^ It remains unclear whether subjective QOL is equally reliable and valid in Japanese culture, given that it is influenced by individual life perspectives, religion, ethnic customs, and values. Hence, this study developed the Japanese version of the BBQ (BBQ‐J) and examined its reliability and validity.

## METHODS

### Translation

Permission to create the BBQ‐J was obtained from the creator of the original BBQ. The BBQ‐J (see the File S1) was translated into Japanese independently by the authors (S.H. and K.M.). Then, a professional translator, who was blinded to the original BBQ, back‐translated the initial Japanese version. After the back translation, we discussed the translations with one of the original authors (G.A.) and confirmed that the original meaning of each item had not changed.

### Participants

An online, cross‐sectional survey was conducted from March 18 to March 23, 2021, with the help of Asmark, Inc. Participation was voluntary, and participants were paid a small monetary reward through the research firm. The protocol for this observational study was reviewed and approved by the Ethics Review Committee of the Graduate School of Medicine, Chiba University (Approval No. 4129). Only complete data, excluding missing data, were delivered by Asmark. A total of 637 Japanese individuals (324 males and 313 females, mean age 35.23 years, SD = 16.88 years) aged between 20 and 87 years were included in our analysis. Table 1 presents the participants' demographics.

**Table 1:** Participants' demographic data (*n* = 637).

Age (years), mean ± SD		35.2 ± 16.9
Work status		
	Full time	208 (32.7%)
	Part time	59 (9.3%)
	Unemployed	88 (13.8%)
	Student	282 (44.3%)
Living status		
	Alone	158 (24.8%)
	With family or a partner	470 (73.8%)
	With others	9 (1.4%)
Household income		
	≤JPY 4,270,000	284 (44.6%)
	>JPY 4,270,000	353 (55.4%)

### Measures

Three self‐report scales were used in this study, including the BBQ. The Satisfaction With Life Scale (SWLS)^18^, ^19^ and the Patient Health Questionnaire‐9 (PHQ‐9) were included to assess convergent validity.^20^, ^21^

#### Brunnsviken Brief Quality of Life Scale

The BBQ measures one's satisfaction with and importance of six life domains: leisure time, view on life, creativity, learning, friends and friendship, and view of self.^5^ The scale comprises 6 × 2 items, with items measuring satisfaction with and importance of each life domain. The items are rated on a five‐point Likert scale ranging from 0 to 4. The total score ranges from 0 to 96 points and is calculated by multiplying the Satisfaction and Importance items for each life domain and summing the six products. The higher the total score, the higher the level of satisfaction with and importance of each life area.

#### Satisfaction With Life Scale

The Satisfaction With Life Scale and Patient Health Questionnaire‐9 were included to assess convergent validity. The SWLS is a scale that measures one's happiness and satisfaction with life.^18^, ^19^ Among the various components of subjective well‐being, it focuses exclusively on assessing overall life satisfaction. It comprises five items that are rated on a seven‐point scale. The total score ranges from 5 to 35 points. The higher the score, the higher the level of satisfaction with life.

#### Patient Health Questionnaire‐9

The PHQ‐9 is a nine‐item scale that assesses depressive symptoms.^20^, ^21^ Each item is rated on a four‐point scale ranging from 0 (*not at all*) to 3 (*nearly every day*). The total score ranges from 0 to 27 points. A total score of 0–4 denotes no symptoms, while scores of 5–9, 10–14, 15–19, and 20–27 denote mild, moderate, moderate‐to‐severe, and severe depressive symptoms, respectively.

### Statistical analysis

All analyses were conducted using the IBM SPSS Statistics package Version 29.0 and AMOS Version 29.0. Data were analyzed as follows: First, descriptive statistics were calculated for each scale, and a *t*‐test was conducted with sex as the independent variable and the BBQ‐J as the dependent variable to examine sex‐based differences. Second, a confirmatory factor analysis was conducted to confirm the factor structure of the BBQ‐J. Goodness‐of‐fit was assessed based on a criterion recommended by Schreiber's (2008) guidelines.^22^ Comparative fit index (CFI) ≥ 0.95, Tucker–Lewis index [TLI] ≥ 0.95, standardized root‐mean‐square residual (SRMR) ≤ 0.08, and root‐mean‐square error of approximation (RMSEA) ≤ 0.06 were used. The original BBQ was explicitly developed to result in a one‐factor structural model. Therefore, we also endeavored to create a one‐factor structural model for 637 people. Additionally, we conducted an exploratory factor analysis to see if there was a better fitting model for the same sample of 637 people. Third, we calculated the Pearson product‐rate correlation coefficient with the SWLS to examine the convergent validity of the BBQ‐J. The SWLS has been used in the field of health psychology to examine the subjective QOL of people with serious health concerns.^23^ We used the SWLS to determine convergent validity. Negative convergent validity was determined by calculating the Pearson product‐rate correlation coefficient with the PHQ‐9. We used Cohen's criteria to determine the degree of correlation, considering |r| ≥ 0.50, |r| ≥ 0.30, and |r| ≥ 0.10 as strong, moderate, and weak correlation, respectively.^24^ Finally, a reliability analysis was conducted to examine internal consistency. Cronbach's alpha of the BBQ‐J was calculated, a benchmark value of ≥0.70 was used as the criterion for acceptable internal consistency.^25^

## RESULTS

### Effect of gender

Table 2 shows the effect of demographics on the BBQ‐J scores. We did not find an effect of sex on the total score of the BBQ‐J and the scores for each life domain after calculating Bonferroni‐corrected *p*‐values.

**Table 2:** Effects of demographics.

	All	Male	Female	Statistics	*p*‐value	*Bonferroni‐corrected p‐value*
	Mean (SD)	Mean (SD)	Mean (SD)			
BBQ Total	35.41 (22.28)	34.58 (23.37)	36.27 (21.09)	*t*(635) = 0.96	0.339	1.000
Leisure	7.04 (4.75)	6.70 (4.79)	7.39 (4.69)	*t*(635) = 1.83	0.067	0.469
View on life	5.91 (4.30)	5.90 (4.43)	5.92 (4.17)	*t*(635) = 0.83	0.934	1.000
Creativity	5.59 (4.34)	5.62 (4.46)	5.56 (4.22)	*t*(635) = 0.16	0.873	1.000
Learning	5.74 (4.29)	5.68 (4.58)	5.81 (3.96)	*t*(635) = 3.82	0.703	1.000
Friends and friendship	5.80 (4.52)	5.43 (4.47)	6.19 (4.56)	*t*(635) = 2.12	0.035*	0.245
View of self	5.36 (4.25)	5.38 (4.42)	5.34 (4.07)	*t*(635) = 0.14	0.889	1.000

Abbreviations: BBQ, Brunnsviken Brief Quality of Life Scale; SD, standard deviation.

* *p*  <  0.05.

### Internal consistency

Cronbach's alpha of the BBQ‐J met the criteria and was sufficient (*α =* 0.92). Inter‐item correlations ranged from *r* = 0.60 to *r* = 0.73, all of which were positive (*p* ≤ 0.001; Table 3).

**Table 3:** BBQ‐J inter‐item correlation matrix Pearson correlation coefficients.

	Leisure	View on life	Creativity	Learning	Friends and friendship	View of self
Leisure		0.68***	0.67***	0.61***	0.60***	0.60***
View on life			0.78***	0.69***	0.63***	0.73***
Creativity				0.73***	0.61***	0.70***
Learning					0.67***	0.68***
Friends and friendship						0.67***
View of self						

*** *p* < 0.001.

### Confirmatory factor analysis

**Figure 1:**
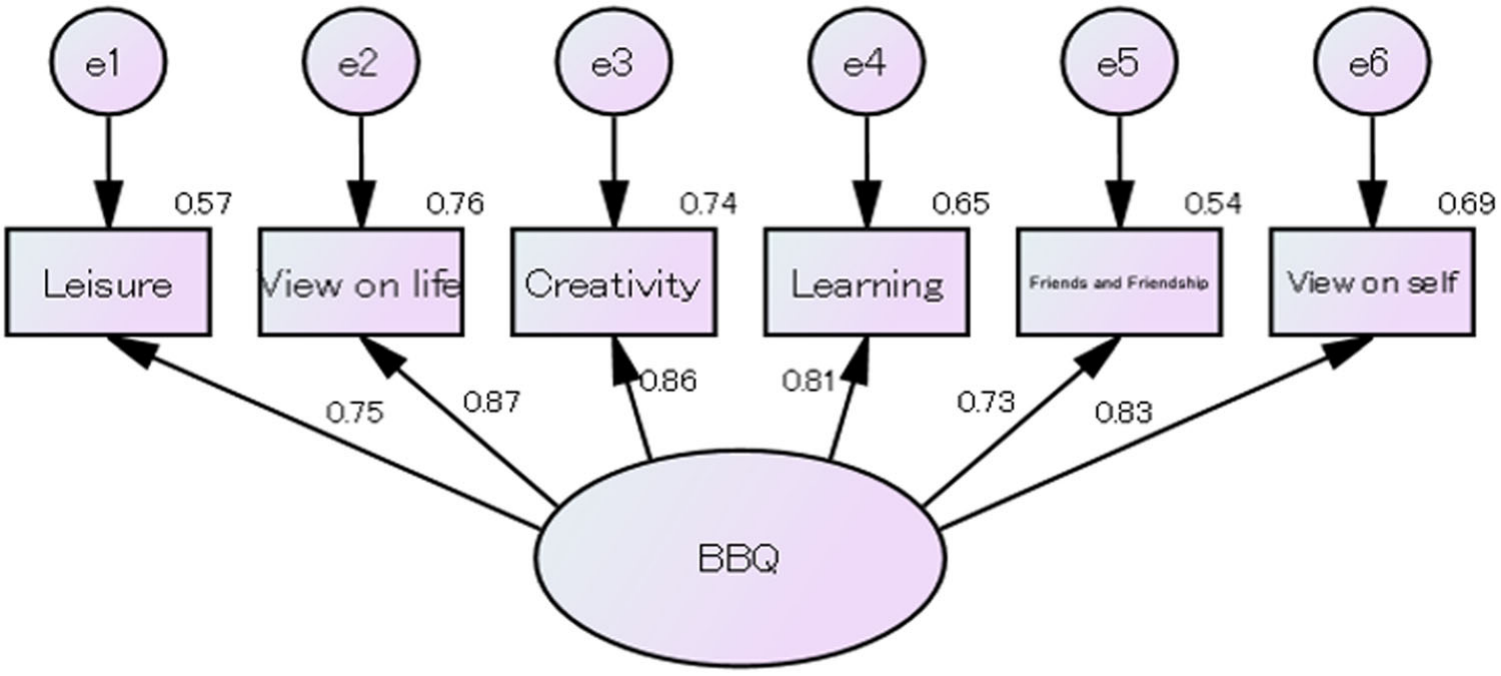
Results of confirmatory factor analysis of the Japanese version of the Brunnsviken Brief Quality of Life Scale (BBQ‐J).

Based on the original BBQ, we constructed a one‐factor model with the items of the BBQ‐J and performed a confirmatory factor analysis to confirm the factor structure of the BBQ‐J. All criteria were met, except for that of RMSEA (CFI = 0.976, TLI = 0.960, SRMR = 0.024, and RMSEA = 0.107). Further, all factor loadings were statistically significant (*p* < 0.001; Figure 1).

### Exploratory factor analysis

**Figure 2:**
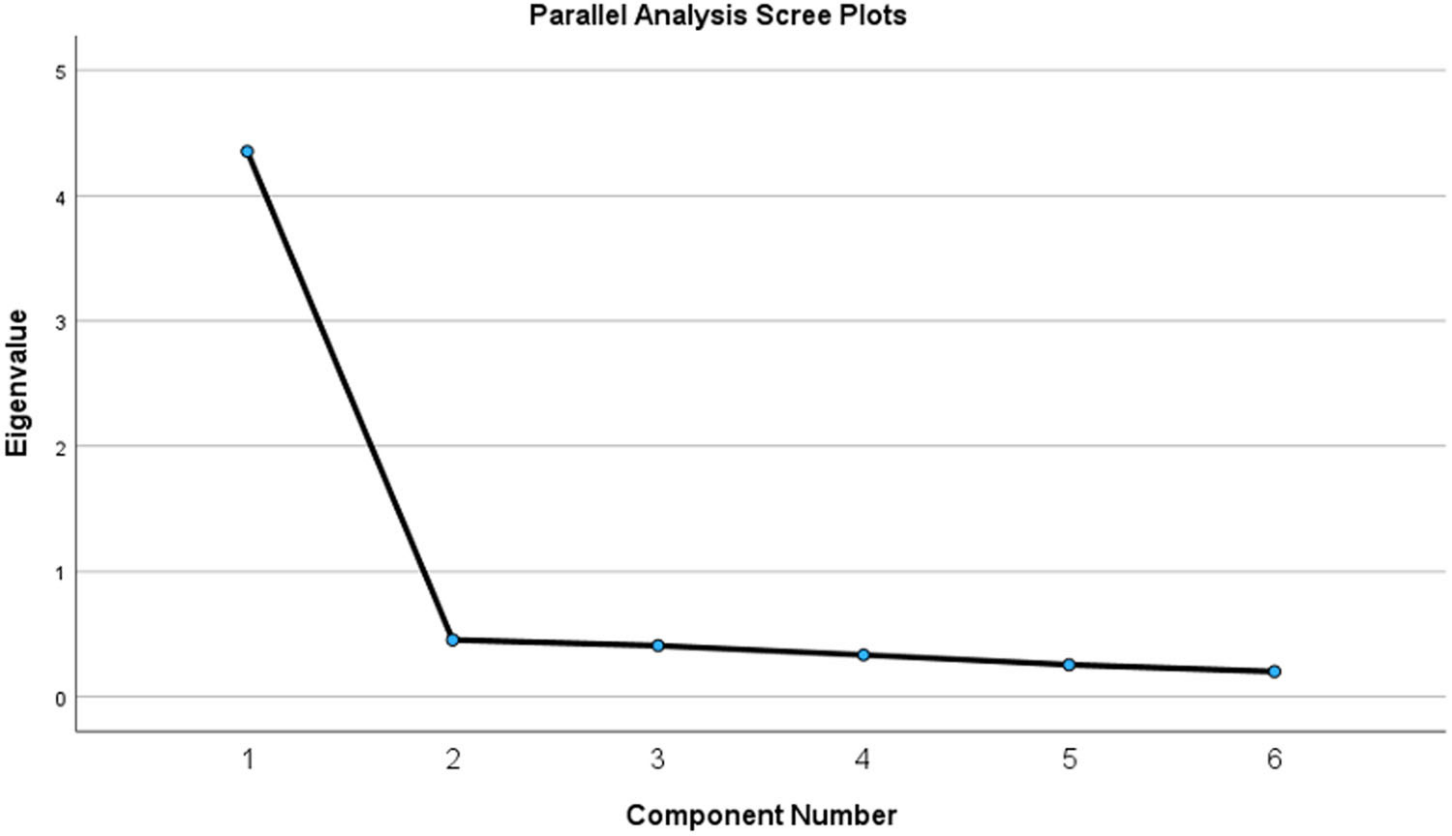
Results of parallel analysis of the Japanese version of the Brunnsviken Brief Quality of Life Scale (BBQ‐J).

A factor analysis using the maximum likelihood method using varimax rotation was performed on the six items. The results are shown in Figure 2. When extracting a single factor, item factor loadings ranged from 0.76 to 0.87 (leisure: 0.76, view on life: 0.87, creativity: 0.87, learning: 0.82, friends and friendship 0.76, and view on self: 0.83).

### Convergent and negative convergent validity of the BBQ‐J

Total scores on the BBQ‐J were positively correlated with total scores on the SWLS (*r* = 0.59, *p* ≤ 0.001). Conversely, total scores on the BBQ‐J were negatively correlated with total scores on the PHQ‐9 (*r* = −0.25, *p* ≤ 0.001).

## DISCUSSION

This study confirmed and validated the factor structure of the BBQ‐J. The results showed that the factor structure of the BBQ‐J was similar to that of the original version and comprised one factor: subjective QOL. We also demonstrated the internal reliability and validity of the BBQ‐J. Although the original version has been validated with respect to standardization,^5^ to the best of our knowledge, this study is the first to formally validate a Japanese‐language version of the scale. This study is also significant because it confirmed the factor structure of the BBQ‐J and validated it as a self‐report measure of subjective QOL.

The scores on the BBQ‐J did not differ significantly between Japanese males and females. This result suggests that, similar to the items of the original BBQ, items of the BBQ‐J were not gender‐biased.^5^ The Cronbach's alpha of the BBQ‐J's subscale was 0.92, which is greater than the Cronbach's alpha of the original BBQ (0.76). This result indicates that the BBQ‐J has good internal consistency for assessing the subjective QOL of Japanese individuals.

The validity of the BBQ‐J was tested by determining its convergent and negative convergent validity, which were determined by examining the scale's scores correlation with the scores on the SWLS and PHQ‐9, respectively. As expected, total scores on the BBQ‐J were positively strong correlated with total scores on the SWLS. Conversely, with respect to negative convergent validity, total scores on the BBQ‐J were negatively weak correlated with total scores on the PHQ‐9. These results indicate that higher scores on the BBQ‐J correlate with a greater degree of satisfaction with health and inversely correlate with mental health problems, such as depression. Overall, these findings indicate that the BBQ‐J is sufficiently accurate for measuring subjective QOL. This result is consistent with that concerning the original BBQ and it can be highly useful for international comparisons. As mentioned earlier, the original BBQ has been shown to identify individuals leading healthier lifestyles,^11^ making it a predictor of both short‐term and long‐term overall health.^12^, ^16^ The BBQ‐J allows for the assessment of subjective satisfaction in the lives of Japanese patients with mental disorders, potentially serving as an indicator of favorable outcomes. These hypotheses should be investigated in future research studies by using the BBQ‐J.

### Limitations

This study has a few limitations. First, Lindner et al.^5^ used the QOL Inventory (QOLI)^26^ to assess convergent validity; however, a Japanese version of the QOLI has not been developed. Therefore, we used the SWLS to determine convergent validity. Although the SWLS is used to examine subjective QOL,^23^ it is different from the scale used in the original study (QOLI). Second, since the study population was the general public and we did not take into account the presence or absence of mental or physical illness, it is necessary to examine the applicability of our findings to a clinical group. Finally, all participants lived in Japan, and the questionnaire was written in Japanese; thus, we assumed that the participants could understand Japanese. However, we were unable to gather information on their cultural backgrounds.

### Conclusions

The BBQ‐J developed in this study has a valid internal structure as a measure of subjective QOL, and it functions similarly to its original version. If the BBQ is available in numerous languages, it will make it possible and easy to compare international studies and conduct international multicenter studies in multiple languages.

## AUTHOR CONTRIBUTIONS

S.H. designed and managed the study, translated the BBQ, analyzed the data, and wrote the initial draft of the manuscript. K.M. designed the study, translated the BBQ, and contributed to the interpretation of the data and revision of the manuscript. E.S. and Y.M. designed the study, contributed to interpretation of the data, and assisted in the preparation of the manuscript. P.L. suggested analyses and contributed to the interpretation of the findings and revisions of the manuscript. G.A. checked the back‐translated BBQ and contributed to the interpretation of the data and revision of the manuscript. All authors read and approved the final manuscript.

## CONFLICT OF INTEREST STATEMENT

The authors declare no conflicts of interest.

## ETHICS APPROVAL STATEMENT

The protocol for this study was reviewed and approved by the Ethics Review Committee of the Graduate School of Medicine, Chiba University (Approval No. 4129).

## PATIENT CONSENT STATEMENT

All participants gave informed consent to participate in the study.

## CLINICAL TRIAL REGISTRATION

N/A.

## Supporting information

Supporting information.

## Data Availability

Like the original BBQ, the BBQ‐J is freely available for non‐commercial use in research and clinical practice and can be downloaded at http://www.bbqscale.com.
