# Supplementary material for: Reliability and validity of a Japanese version of the Brunnsviken Brief Quality of Life Scale
Source: PCN Rep. 2024 Feb 4;3(1):e170. doi: 10.1002/pcn5.170 (PMC11114274; doi:10.1002/pcn5.170)
Supplement: Supplementary file 1 — Supporting information. [file PCN5-3-e170-s001.pdf]

生活満足度尺度（The Brunnsviken Brief Quality of Life Scale - Japanese version, BBQ-J）

次の 12 の質問は、あなたがどのように生活の質を体験しているかに関するものです。これは 6 つの領域をカバーしており、これらにどれだけ満足しているのか、そしてそれらがあなたにとってどれほど重要なものなのかが書かれています。あなたの経験を最もよく反映している数字に〇をつけてください。

		**まったく同意しない**				**完全に同意する**
1	私は、**自分の余暇**に満足しています。リラックスして楽しむために、自分の好きなことをする機会があります。	0	1	2	3	4
2	**余暇**は、私の生活の質を高めるために大切なものです。	0	1	2	3	4
3	私は、**自分の人生観**に満足しています。私は自分にとって何が意味があり、何を信じ、そして自分の人生で何をしたいのかを知っています。	0	1	2	3	4
4	**自分の人生をどう捉えるか**は、私の生活の質にとって大切なことです。	0	1	2	3	4
5	私は、日常生活、趣味、仕事、勉強などで想像力を働かせ、**クリエイティブ**でいる機会に満足しています。	0	1	2	3	4
6	**クリエイティブ**でいられることは、私の生活の質を高めるために大切なことです。	0	1	2	3	4
7	私は、**学び**に満足しています。私には、興味を引く新しいワクワクするようなことやスキルを学ぶ機会と欲求があります。	0	1	2	3	4
8	**学び**は、私の生活の質にとって重要です。	0	1	2	3	4
9	私は、**友人と友人関係**に満足しています。私には、付き合いのある友人や、支えてくれる友人がいます（私には必要で欲しいだけの友人がいます）。	0	1	2	3	4
10	**友人と友人関係**は、私の生活の質にとって重要です。	0	1	2	3	4
11	私は、**1人の人間としての私**に満足しています。私は自分が好きでリスペクトしています。	0	1	2	3	4
12	**1人の人間としての自分**に対する私の満足度は、私の生活の質にとって重要です。	0	1	2	3	4

研究者や臨床家は BBQ を無料で自由に使用できます。

詳細については、www.bbqscale.com をご覧ください。
